# Chelonid Alphaherpesvirus 5 Prevalence and First Confirmed Case of Sea Turtle Fibropapillomatosis in Grenada, West Indies

**DOI:** 10.3390/ani11061490

**Published:** 2021-05-21

**Authors:** Amanda James, Annie Page-Karjian, Kate E. Charles, Jonnel Edwards, Christopher R. Gregory, Sonia Cheetham, Brian P. Buter, David P. Marancik

**Affiliations:** 1 Department of Pathobiology, School of Veterinary Medicine, St. George’s University, True Blue, Grenada; ajames5@sgu.edu (A.J.); jedward6@sgu.edu (J.E.); scheetha@sgu.edu (S.C.); bbutler@sgu.edu (B.P.B.); 2 Harbor Branch Oceanographic Institute, Florida Atlantic University, Fort Pierce, FL 34946, USA; cpagekarjian@fau.edu; 3 Ocean Spirits, Inc., Grand Anse, Grenada; kate@oceanspirits.org; 4 Infectious Diseases Laboratory, University of Georgia, Athens, GA 30602, USA; crg@uga.edu

**Keywords:** sea turtle, fibropapillomatosis, chelonid herpesvirus-5

## Abstract

**Simple Summary:**

Fibropapillomatosis is a disease of sea turtles that is likely caused by the virus chelonid alphaherpesvirus-5. Sea turtles, and especially green sea turtles, can develop extensive tumors that impede foraging and swimming and can result in high morbidity and mortality. The presence of the virus has not been assessed in Grenada and fibropapillomatosis has anecdotally not been observed in the island’s sea turtle aggregates. From 2017 to 2019, a total of 167 green, leatherback, and hawksbill turtles were examined for fibropapilllomatosis. Skin and blood samples were examined for the presence of the virus and previous exposure to the virus was assessed by checking for antibodies in the blood. No signs of fibropapillomatosis or active viral infection were found in any turtle examined during the study. Antibody testing showed that 34.6% of green turtles examined had been previously exposed to the virus. In 2020, the first case of fibropapillomatosis occurred in a green turtle in Grenada and the presence of the virus was confirmed in tumor samples. These results indicate that to date, active viral infection is rare in Grenada’s turtles, although viral exposure in green sea turtles is relatively high. The impact of fibropapillomatosis in Grenada is suggested to be low at the present time, and further studies examining factors that may influence disease are warranted.

**Abstract:**

Chelonid alphaherpesvirus 5 (ChHV5) is strongly associated with fibropapillomatosis, a neoplastic disease of sea turtles that can result in debilitation and mortality. The objectives of this study were to examine green (*Chelonia mydas*), hawksbill (*Eretmochelys imbricata*), and leatherback (*Dermochelys coriacea*) sea turtles in Grenada, West Indies, for fibropapillomatosis and to utilize ChHV5-specific PCR, degenerate herpesvirus PCR, and serology to non-invasively evaluate the prevalence of ChHV5 infection and exposure. One-hundred and sixty-seven turtles examined from 2017 to 2019 demonstrated no external fibropapilloma-like lesions and no amplification of ChHV5 DNA from whole blood or skin biopsies. An ELISA performed on serum detected ChHV5-specific IgY in 18/52 (34.6%) of green turtles tested. In 2020, an adult, female green turtle presented for necropsy from the inshore waters of Grenada with severe emaciation and cutaneous fibropapillomas. Multiple tumors tested positive for ChHV5 by qPCR, providing the first confirmed case of ChHV5-associated fibropapillomatosis in Grenada. These results indicate that active ChHV5 infection is rare, although viral exposure in green sea turtles is relatively high. The impact of fibropapillomatosis in Grenada is suggested to be low at the present time and further studies comparing host genetics and immunologic factors, as well as examination into extrinsic factors that may influence disease, are warranted.

## 1. Introduction

Grenada, a multi-island country in the Windward Islands of the Caribbean Sea, supports nesting and foraging aggregations of endangered green turtles (*Chelonia mydas*), critically endangered hawksbills turtles (*Eretmochelys imbricata*), and nesting aggregations of vulnerable leatherback turtles (*Dermochelys coriacea*) [1]. These populations are believed to be in decline consistent with worldwide trends as six of the seven species of sea turtles distributed globally are listed as vulnerable, endangered, or critically endangered by the International Union for Conservation of Nature (IUCN) [2]. 

There is a growing amount of literature describing infectious disease threats in sea turtles. Multiple diseases have been attributed to viruses from the *Herpesviridae* family, which have been documented in all species of sea turtles [3,4,5,6,7,8,9]. This includes grey-patch disease, lung-eye-trachea disease, and fibropapillomatosis [10]. Of these diseases, fibropapillomatosis is arguably the most significant due to its worldwide distribution, prevalence, and disease severity [11].

Fibropapillomatosis presents clinically as cutaneous fibropapillomas and internal fibromas, fibrosarcomas, and myxofibromas [12,13,14,15]. As the disease progresses the tumors may increase in size and number, and negatively affect the turtle’s ability to swim, forage, and escape predation, which in severe cases can result in debilitation and death [16]. If the turtle can compensate for the disease or receives veterinary intervention, the tumors may spontaneously regress.

There is a strong association between fibropapillomatosis and the presence of chelonid alphaherpesvirus 5 (ChHV5). Early ultrastructural studies of lesions visualized herpesvirus-like particles within tumor cells [17,18,19], suggesting their association with a viral agent. Since that time, ChHV5 DNA has been characterized molecularly from diseased tissues of affected turtles, and less often in non-diseased tissues of turtles [20,21,22]. The virulence of ChHV5 has been demonstrated in vitro in green turtle skin cells [23] and the transmission of disease has been achieved using cell-free tumor extracts [24]. However, to date, Koch’s postulates have not been completely fulfilled for ChHV5 due to challenges associated with culturing the virus.

The relationship between ChHV5 DNA and the formation of tumors is not clear, as multiple studies have found that clinically healthy turtles can carry the virus [21,22,25]. There may be genetic or environmental components that influence disease prevalence rates within a given population of turtles [24]. Tumors are more likely to be observed in juvenile sea turtles than in adults [26,27], suggesting a biological or behavioral component to the disease. Another consideration is the characteristic latency of herpesvirus infections, as infected animals can go extended periods of time with undetectable levels of the virus until the pathogen is reactivated due to host stress, immunosuppression, or age, resulting in morbidity [28]. The degree to which ChHV5 follows traditional herpesvirus pathogenesis is unknown, as the clinical disease of fibropapillomatosis has not been described in other vertebrates with herpesvirus infection.

Evaluating cases of fibropapillomatosis has provided some insight into how the disease affects various sea turtle species worldwide. Previous studies using globally sourced samples suggest overall ChHV5 prevalence may be as high as 100% among tumor-exhibiting green turtles, and 15% in clinically healthy leatherback, hawksbill, loggerhead (*Caretta caretta*), and olive ridley turtles (*Lepidochelys olivacea*) [22]. Despite this generalized distribution, the virus is only considered to be epizootic in green turtles due to their apparent predisposition to a relatively high and widespread manifestation of clinical disease [13,14,15,16]. ChHV5 genomic sequences amplified from tumors show the global distribution of the virus [11,23], but with significant phylogeographical strain variants [29,30,31,32,33] that have been associated with individual foraging grounds [32,33]. This suggests the majority of viral transmission between sea turtles occurs horizontally. However, sea turtles’ migratory lifestyle and the possible transmission of the virus from clinically healthy turtles complicates epidemiologic tracing [23,27]. 

Prior to 2020, there had been no confirmed reports of fibropapillomatosis in sea turtles in Grenada. However, fibropapillomatosis has been identified in sea turtle aggregates in nearby Venezuela [16], Nicaragua, and Costa Rica [27]. This suggests that nesting and foraging sea turtle aggregates in Grenada may have been exposed to, or are carrying, ChHV5, even if clinical disease is rare or absent. In an effort to better understand whether the lack of observed fibropapillomatosis in Grenada’s sea turtles was a result of limited viral exposure or an absence of disease manifestation, this study utilized a combination of PCR and serology to examine ChHV5 prevalence in green, hawksbill, and leatherback turtles. Elucidation of the prevalence of herpesviruses and ChHV5 in Grenadian turtles will provide further epidemiologic information on this virus and the risks of fibropapillomatosis in the West Indies.

## 2. Materials and Methods

### 2.1. Health Assessment and Sample Collection

The protocols and procedures of this study were approved by the St. George’s University Institutional Animal Care and Use Committee (IACUC-16017-R). Collection and CITES export and import permits were obtained from the Grenada Ministry of Agriculture, Forestry, Lands, and Fisheries and the United States Fish and Wildlife Service (17US34054C/9 and 18US45805C/9). 

Gross examination and sample collection were performed in green and hawksbill turtles from June through August 2017–2019 (Table 1). The sample population is interpreted to consist of immature and mature turtles based on regional descriptions of turtle size at maturation [26,34,35], although the reliable aging of turtles is hindered by a lack of growth curve data specific to Grenada. Turtles were captured by free divers within foraging grounds off the northern and western coasts of Grenada and immediately transferred onto an awaiting boat. Each turtle was examined for external tumors and Monel flipper tags were placed and/or recorded. Each turtle was measured for curved notch-to-tip carapace length (CCL) and carapace width (CCW). Mean CCL and CCW and standard deviation (SD) were calculated for each species (Table 1), although a complete set of samples (i.e., whole blood, plasma, skin) was not always recovered from each turtle.

Blood was collected from the dorsal cervical sinus using the aseptic technique [36]. Each turtle was manually restrained in a prone position, with the head pointed down and the neck extended using gentle traction. The venipuncture site was disinfected with 5% betadine prior to and after blood sample collection. Using a 22-gauge, 1-inch needle, 3 mL of blood was collected into a 3 mL syringe coated with sodium heparin (Kendall Monoject, Mansfield, MA). The blood was immediately transferred into a lithium heparin tube (Greiner Bio-One, Monroe, NC, USA) and placed on ice until transportation to the laboratory within 8 h. Blood samples were aliquoted into approximately 500 µL portions and half the samples were stored as whole blood samples at −80° until DNA extraction. The remaining blood was centrifuged at 5000 rpm for 5 min and plasma to be used for serologic testing was removed and stored at −80°.

Skin biopsies were taken from the interdigital webbing of the hind flipper. The area was disinfected with 5% betadine and straight Metzenbaum scissors were used to remove a 0.5–1.0 cm × 0.5–1.0 cm full-thickness skin sample from the distal portion of the flipper. The skin samples were transferred to individual 1.5 mL microcentrifuge tubes and placed on ice until transportation to the laboratory, which occurred within 8 h of collection. The samples were frozen at −80 °C until DNA extraction.

The blood and skin sampled were collected from adult female nesting leatherback sea turtles in 2017–2018 (Table 1) from March through July on Levera Beach, Grenada, which is the island index nesting beach. Each turtle was examined for external tumors. Monel flipper tags with turtle identification numbers and passive integrated transponder (PIT) tags were placed on the rear flippers and front right shoulder, respectively, or recorded for each female. Morphometric measurements were collected as described above. Blood samples were drawn from the femoral rete system during oviposition [37] and processed and preserved as described for green and hawksbill turtles.

Skin biopsies were taken from the left shoulder of leatherback females. This anatomic site differed from that used for green and hawksbill turtles based on restraint technique and turtle size. The area was disinfected with 5% betadine, and a 6 mm punch biopsy (MedEx Supply, Passaic, NJ, USA) was used to sample the epidermis, the dermis, and the subcutis. After biopsy, pressure was applied to the site until the end of ovipositioning and betadine was re-applied to the area. The biopsy samples were transferred into 1.5 mL microcentrifuge tubes and placed on ice until they were transported to the laboratory within 10 h and stored at −80 °C until DNA extraction.

### 2.2. DNA Extraction and Polymerase Chain Reaction Assay (PCR)

The extraction of total genomic DNA (gDNA) from blood and tissue samples was performed using DNeasy Blood and Tissue Kits (Qiagen, Hilden, Germany) following manufacturer protocols, with the recommended 10 μL of anticoagulated whole blood or 25 mg of epidermal/dermal tissue. Nucleic acid quantity and purity were measured using a NanoDrop Spectrophotometer (Thermoscientific) [38].

Quantitative PCR was conducted at Florida Atlantic University’s Harbor Branch Oceanographic Institute (Fort Pierce, FL USA) using a hydrolysis probe-based assay targeting the UL30 gene segment [39] and a sea turtle beta-actin gene segment (GenBank AY373753.1) as an internal control. UL30 gene segments primers consisting of a forward primer (5′-AAC-GCT-TGC-TTT-TGG-ACA-AG-3′), reverse primer (5′-CCA-GCG-GGT-GTG-AAT-AAA-AT-3′), and hydrolysis probe (5′-6′-FAM-TGG-CCA-TCA-ZEN-AGCTGA-CGT-GCA-3′) for ChHV5 UL30 were used on all samples.

For a wider analysis of herpesvirus infection, degenerate primers amplifying a broad spectrum of conserved herpesvirus DNA polymerases across multiple species were applied in a nested PCR analysis [40] at the University of Georgia, Infectious Disease Laboratory (Athens, Georgia USA) using upstream primers DFA (5′-GAYTTYGCNAGYYTNTAYCC-3′) and ILK (5′-TCCTGGACAAGCAGCARNYSGCNMTNAA-3′) and downstream primer KG1 (5′-GTCTTGCTCACCAGNTCNACNCCYTT-3′). These were run with an internal primer set consisting of upstream primer TGV (5′-TGTAACTCGGTGTAYGGNTTYACNGGNGT-3′) and downstream primer IYG (5′-CACAGAGTCCGTRTCNCCRTADAT-3′).

PCR-positive products were subjected to Sanger sequencing (Genewiz, Plainfield, NJ, USA). The resulting trace files were inspected and trimmed using FinchTV 1.4.0 chromatogram viewing software (Geospiza, Inc., Seattle, WA, USA) and sequences were compared to herpesvirus sequences available in the National Center for Biotechnology Information (NCBI) GenBank sequence repository using the nucleotide Basic Local Alignment Search Tool (BLASTn) database. 

### 2.3. Serology for ChHV5-Specific IgY

To evaluate exposure to ChHV5, serology was performed to detect antibodies to ChHV5 antigens. Sodium chloride was added to plasma samples for defibrination and transformation to serum [41]. Serum was examined using a commercial ELISA test developed and performed at the University of Georgia, Infectious Disease Laboratory (Athens, GA, USA) [42]. The assay was based on a monoclonal anti-turtle IgY validated for all sea turtle species and a synthesized HerbstFibropapGlyh4 peptide (CKALKSGKIEGEDRK) used as antigen [43]. Samples were analyzed in triplicate and with a positive and negative control. Curved carapace length measurements were checked for normal distribution and a Student’s unpaired t-test was used to compare turtle size between seropositive and seronegative turtles.

### 2.4. Gross Necropsy of Green Turtle Mortality

In June 2020, a female green turtle that was covered in numerous fibropapilloma-like growths was found dead in 3–4 m of water, approximately 150 m offshore of BBC Beach, St. George’s, Grenada. The turtle was interpreted to be an adult based on the presence of mature gonads at necropsy. There were no sightings of a debilitated or sick turtle in the area prior to the turtle being found dead. The carcass was transported to St. George’s University School of Veterinary Medicine for necropsy. Morphometrics were recorded and a complete necropsy was performed. Representative samples of all tissues and lesions were preserved in 10% formalin for histopathology. Tissues were routinely processed, sectioned at 5 µm, stained with hematoxylin and eosin, and examined under light microscopy. Lesion-free skin from the left rear flipper and representative tumor samples were preserved in 90% ethanol and processed for ChHV5-qPCR as described above.

## 3. Results

A total of 167 live, free-ranging, green, hawksbill, and leatherback turtles were evaluated for this study between 2017and 2019 (Table 1). There were no external tumors observed (Table 1) in any turtle. Similarly, no ChHV5 or herpesviral DNA was detected in whole blood or non-tumorous skin samples from green, hawksbill, or leatherback turtles (Table 1). ChHV5-specific IgY antibodies were identified in serum samples from 18/52 (34.6%) green turtles, but not in samples from hawksbill or leatherback turtles (Table 1). The mean CCL n-t for seropositive and seronegative green turtles were not significantly different at 47.2 cm ± 13.2 sd and 41.0 cm ± 9.7 sd, respectively. 

### Green Turtle Necropsy

**Figure 1:**
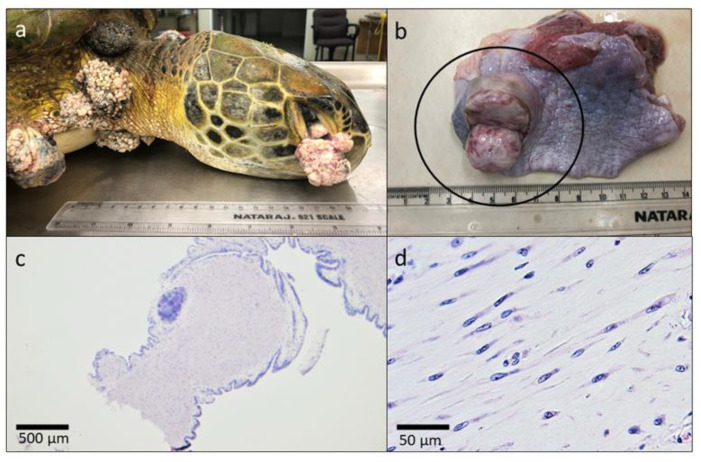
Necropsy findings of the first confirmed case of fibropapillomatosis in Grenada in a green turtle (*Chelonia mydas*). Gross appearance of (**a**) fibropapillomas extending from the conjunctiva of the eye, the neck, and the shoulder and (**b**) incised fibroma within the parenchyma of the lung. Tumor histomorphology under (**c**) 2× magnification demonstrating pedunculated appearance and (**d**) 40× magnification showing well differentiated spindle cells separated by an abundant fibrovascular stroma.

A green turtle carcass that was found dead on BBC Beach, Grenada, had mild post-mortem autolysis and was severely emaciated with a markedly concave and easily depressible plastron, minimal to absent fat deposition within the coelomic cavity, and mild diffuse skeletal muscle atrophy. The curved carapace length was 78.2 cm and CCW was 72.4 cm. There were approximately 31 cutaneous fibropapillomas covering the proximal flippers, neck, shoulders, inguinal and axillary regions, and tail base (Figure 1). They were also present bilaterally on the conjunctiva of the eyes and extended in front of the cornea (Figure 1a). Fourteen of these growths were in excess of 4 cm in diameter for a tumor score of 3 (1–3) [44]. Internally, there was a 3 cm in diameter focal fibroma within the parenchyma of the right cranial lung (1b). The histomorphologic features were similar for all tumors examined and consisted of expansive, pedunculated fibropapillomas comprising well-differentiated spindle cells separated by an abundant fibrovascular stroma (Figure 1c,d). The overlying epithelium was covered with a thick layer of orthokeratotic hyperkeratosis. There were multifocal aggregates of heterophils, lymphocytes, and plasma cells, and scattered vascular thrombi within tumors.

Genomic DNA from three skin tumors and a skin biopsy from a section of grossly unaffected left rear flipper was submitted to the University of Georgia, Infectious Disease Laboratory for qPCR and sequencing as described above. All samples amplified ChHV5 DNA (GenBank accession number MZ048936) that demonstrated >99% identity and 99% coverage to 35 previously submitted ChHV5 sequences in GenBank.

## 4. Discussion

Fibropapillomatosis represents an important disease in sea turtles worldwide, including in the Caribbean Sea [11]. The goals of this study were to examine green, hawksbill, and leatherback turtles in Grenada for signs of fibropapillomatosis and to determine the prevalence of and exposure to ChHV5. Whole blood and skin biopsies were examined by PCR, as viral DNA-emia likely represents an important part of the ChHV5 pathogenesis during primary and reactivated infections [39], and the shedding of virally infected epidermal cells may contribute to new infections [21,26], respectively. Serology for the presence of ChHV5-specific antibodies was examined as previous research indicates a strong positive correlation between antibody reactivity to herpesvirus inclusions and fibropapillomatosis [45]. Additionally, ChHV5 may become latent, making it difficult to detect viral particles in clinically healthy but infected animals [46]. Serologic data also provide evidence of previous ChHV5 infection in clinically recovered animals.

All of the turtles sampled from 2017 to 2019 appeared clinically healthy with no external lesions suggestive of fibropapillomatosis. Consistent with gross observations, whole blood and skin samples were negative for herpesvirus DNA, suggesting a lack of active infection. However, serology demonstrated that close to 35% of green turtles had been previously exposed to ChHV5, suggesting they may be persistently infected. In 2020, the first confirmed case of ChHV5-associated fibropapillomatosis was found in a green turtle in Grenada. These results indicate that infection is occurring within Grenada’s inshore green turtle aggregates, although clinical manifestation of the disease is rare.

The absence of ChHV5 DNA-positive turtles in the surveillance portion of this study was unexpected. The molecular detection of ChHV5 DNA in blood and skin samples taken from free-ranging sea turtles in other geographic regions suggests infection rates between 15% and 100%, depending on species and location, with green turtle prevalence ranging in the higher percentages [22]. Additionally, the testing of skin from green turtles with no visible signs of fibropapillomatosis has demonstrated variable ChHV5 prevalence rates between 5.2 and 60% depending on geographic region and the type of assay used [47,48,49]. The 55 blood samples and 49 skin samples from green turtles analyzed herein provide enough statistical power to detect ChHV5 DNA at a 95% confidence level if the prevalence of ChHV5 is 5% or more within the population [50], conservatively assuming that the sensitivities of the molecular assays are 95%. Similarly, the number of hawksbill and leatherback turtles sampled should provide 95% confidence of detection at an assumed pathogen prevalence of approximately 10% or higher. This indicates that active infection rates are relatively low in Grenada compared to worldwide data. The positive serology results indicate that the rare occurrence of fibropapillomatosis in green turtles in Grenada is not due to a lack of exposure to the virus, while this may be true for hawksbill and leatherback turtles.

Although none of the turtles sampled between 2017 and 2019 tested positive for ChHV5 DNA, it is possible they could have tested positive at other anatomic locations that were not examined. The distribution and duration of ChHV5 in fluids and tissues during the course of infection is unknown. Detectable loads of ChHV5 DNA within blood samples from other studies suggest infected turtles go through periods of viremia [39,45]. ChHV5 DNA-emia was not observed in any of the blood samples analyzed in this study, suggesting that these turtles tested were not systemically infected. There is a substantial amount of data demonstrating that ChHV5 has a predilection for dermal tissue [17,18,19,20,21,22]. However, in one study, ChHV5 DNA was detected in only 47.6% of normal skin samples taken from the shoulder region of turtles that had tumors in other areas of the body [21]. This demonstrates that ChHV5 is not present diffusely throughout the skin of infected turtles. Another important consideration is the latency periods traditionally seen with herpesvirus infections [28]. Latency has not yet been described with ChHV5, but repeat sampling of the same turtle may be useful in evaluating infection status trends over time, especially in PCR-negative and seropositive turtles. It may also be beneficial to increase the number of sites sampled. ChHV5 has been detected in a wider range of ante-mortem sample types than those measured in this study. For example, ocular, nasal, and cloacal swabs have tested positive for ChHV5 using molecular methods [27]. Demonstrated differences in sensitivity between primer sets also indicate that the use of multi-gene assays can optimize the detection of infection [31,48,51].

It is unknown if the confirmed case of fibropapillomatosis in 2020 is a resident turtle or if it had recently migrated into inshore waters, as it was untagged. The migratory routes of nesting and foraging turtles found in Grenada have not been well characterized, but they likely migrate from numerous regions within and outside of the Caribbean. For example, in a study following green turtles tagged in Tortuguero, Costa Rica, 3833 turtles were recaptured in Nicaragua, 30 in Venezuela, 1 in Puerto Rico, and 1 in Grenada [52]. Hawksbill turtles that breed in Barbados migrate to foraging grounds in Grenada [53] and are composed of mixed genetic lineages from other areas, including Florida, USA and Costa Rica [54]. As fibropapillomatosis and ChHV5 have been documented in these countries [16,27,55], it is not surprising that infected turtles reach Grenada on their migration routes. More studies are warranted to further characterize viral exposure and population dynamics in Grenadian turtles. This includes genotyping turtles to examine if there is an association between antibody serostatus and natal origin. 

The low incidence of active ChHV5 infection and fibropapillomatosis in Grenada is likely affected by a number of interactions between the host, pathogen, and environment. This may include host susceptibility to infection, as clinical disease in non-*C. mydas* species is rare. Natural, family-based pathogen resistance has been described in many groups of animals, including fishes, reptiles, and mammals [56,57,58]. It is possible that innate immunity impacts infection rates and disease progression, although hereditary effects and immune response to ChHV5 have not been well explored in sea turtles. Host age may also influence ChHV5 infection and fibropapillomatosis development. As mentioned previously, fibropapillomatosis is more commonly observed in juvenile turtles compared to adults [26,27]. The 64.8 cm and 44.9 cm mean CCL for hawksbill and green turtles, respectively, trended towards measurements commonly described for adult hawksbill turtles and juvenile and subadult green turtles [26,34,35]; however, the size at which hawksbill and green turtles reach maturity in Grenada has not been described, and the ages of turtles sampled cannot be reliably estimated until regional growth curves have been established. The present study only included samples from adult female leatherback turtles, as leatherback foraging grounds are not found locally due to their pelagic life history. If ChHV5 is present in Grenada, but at prevalence levels lower than 5–10% depending on species, targeting at-risk juvenile turtles may increase the probability of detecting ChHV5.

The absence of detected ChHV5 in sea turtles in Grenada may also be associated with environmental conditions. There are multiple environmental influences that have been associated with tumor proliferation in green turtles, and the process is likely multifactorial. Green turtles with severe tumor burdens have been found to contain significantly higher concentrations of exogenous metals and lipid peroxidation in their blood compared to turtles with mild or absent clinical disease [59]. The authors suggest that oxidative damage caused by metal contamination may cause immunosuppression leading to more pronounced disease. The lack of observed gross lesions in Grenada’s turtles may suggest that relatively lower environmental stressors exist regionally compared to more industrialized coastal regions. This hypothesis is supported by studies that describe relatively low environmental anthropogenic pressure compared to other Caribbean islands [60,61,62].

In a study on environmental eutrophication in Hawaii, the prevalence of clinical fibropapillomatosis paralleled nitrogen-footprint patterns associated with urbanization, sewage injection wells, sugar and pineapple agriculture, and boat harbors [63,64]. One sequela that can occur with these events is nutrient overload, which may result in increased algal blooms [65]. Oncogenic compounds such as okadaic acid, lyngbyatoxin A, brevetoxins, and other biotoxins produced by algal species have been hypothesized to be associated with fibropapillomatosis [66,67,68]; however, no correlation has been found between fibropapillomatosis and toxin exposure [69]. The role this plays in the pathogenesis of fibropapillomatosis is unknown, although in general, turtle health is closely linked to the health of the environment [70]. The environmental conditions associated with Grenada’s relatively unaffected turtle populations may offer comparisons to regions with higher pathogen and disease prevalence. Elucidating these baseline environmental parameters becomes important as inshore areas surrounding Grenada remain at risk ecologically [71], and changes in environmental conditions could have direct implications for turtle health. Future studies examining toxicologic levels of metals, other contaminants, and immunologic markers in sea turtles in Grenada are warranted.

## 5. Conclusions

This study was the first to document fibropapillomatosis and examine ChHV5 prevalence in sea turtles in Grenada. These findings indicate that at this time, the impact of fibropapillomatosis on Grenadian turtle aggregates is minimal, although the virus and disease are present in green turtles. This provides a foundation for future research into proposed host, pathogen, and environmental factors that may affect viral transmission. Baseline studies comparing the population genetics and immunological characteristics of Grenadian turtles to aggregates in areas with a high incidence of infection and disease are indicated. A further understanding of this disease is crucial for endangered sea turtle populations and our ability to develop effective conservation strategies.

## Figures and Tables

**Table 1:** Morphometric measurements and herpesvirus testing data for green (*Chelonia mydas*), hawksbill (*Eretmochelys imbricata*), and leatherback turtles (*Dermochelys coriacea*) in Grenada, West Indies.

Species	Mean CCL ± SD Mean CCW ± SD (cm)	CCL Range (cm)	Blood ChHV5 qPCR and Herpesvirus nPCR	Skin ChHV5 qPCR and Herpesvirus nPCR	Serology
Green turtles (*n* = 56)	44.9 ± 11.1 39.3 ± 10.0	29.8–65.3	0/55 (0%)	0/49 (0%)	18/52 (34.6%)
Hawksbill turtles (*n* = 53)	64.8 ± 20.1 55.4 ± 18.1	31.1–95.8	0/36 (0%)	0/34 (0%)	0/22 (0%)
Leatherback turtles (*n* = 58)	152.9 ± 7.2 112.2 ± 9.9	110–167.8	0/38 (0%)	0/40 (05)	0/29 (0%)

CCL = notch to tip curved carapace length; CCW = curved carapace width; SD = standard deviation; ChHV5 = chelonid alphaherpesvirus 5; qPCR = quantitative polymerase chain reaction; nPCR = nested polymerase chain reaction, SD = standard deviation.

## Data Availability

All relevant data are within the manuscript, and are fully available without restriction.
